# Severe cervical kyphosis in a complex child with NF1, case report and literature review

**DOI:** 10.1007/s00381-025-06831-3

**Published:** 2025-05-10

**Authors:** Luigi Aurelio Nasto, Ferruccio De Prisco, Enrico Pola, Silverio Perrotta, Giuseppina Miele, Gianluca Piatelli, Claudia Santoro

**Affiliations:** 1 Department of Orthopaedics, “Luigi Vanvitelli” University Hospital, University of Campania “Luigi Vanvitelli”, Via del Sole 10, 80138 Naples, Italy; 2 Department of Women’s and Children’s Health and General and Specialized Surgery, University of Campania “Luigi Vanvitelli, ” Via Luigi de Crecchio 2, 80138 Naples, Italy; 3 Department of Advanced Medical and Surgical Sciences, University of Campania “Luigi Vanvitelli”, 80131 Naples, Italy; 4 Department of Neurosurgery, IRCCS Istituto “G. Gaslini” Children’s Hospital, Genoa, Italy

**Keywords:** Neurofibromatosis type 1, Kypho-scoliosis, IMEK, Plexiform neurofibroma

## Abstract

**Purpose:**

We faced and herein report a detailed description of pre-operative assessment, management, and post-operative follow-up of a 2-year and 10-month-old girl with neurofibromatosis 1 (NF1) who presented with severe, dystrophic, cervical kyphosis (170 degrees) associated with extensive pre- and para-vertebral plexiform neurofibromas, who also went under MEK inhibitors therapy. Cervical kyphosis in NF1 is particularly rare, and there is no extensive literature available on the subject in terms of clinico-radiological features, surgical approach, and outcomes. We therefore also performed a comprehensive review of the available literature on the topic.

**Methods:**

The clinical report was made through the retrospective review of all medical documents and imaging of the patient. The systematic review was performed based on the inclusion and exclusion criteria set by the authors on surgical management of cervical kyphosis in NF1 patients according to the Preferred Reporting Items for Systematic Reviews and Meta-Analyses (PRISMA).

**Results:**

Our patient underwent a first-stage halo-gravity traction followed by a single-stage occipito-cervical posterior fusion. The six-week traction resulted in a reduction of the deformity from 170 to 90°. A further amelioration was obtained by surgery with a final 60% correction of the curvature (69° at last post-operative X-ray). No complications were observed at 1-and-a-half-year follow-up. The plexiform neurofibromas were treated with MEK inhibitors: trametinib for 1 year and 11 months until performing halo traction, and with selumetinib after surgery. We just found 19 papers suitable according to our selection criteria.

**Conclusion:**

Combined anterior and posterior fusion (CAP) is generally the best treatment option, although it is not always feasible. When plexiform, symptomatic, inoperable neurofibromas coexist, surgery can be preceded or followed by MEK inhibitor treatment for better control or a volumetric reduction of the tumors. The best therapeutic choice should always be the result of a multidisciplinary, expert approach and patient-tailored design.

## Introduction

Neurofibromatosis type 1 (NF1) is an autosomal dominant, multi-system disorder that predominantly involves the central and peripheral nervous systems and is associated with an increased risk of tumor development [1]. Bone abnormalities are also common, particularly in pediatric patients, with sphenoid dysplasia or long bone dysplasia, which is considered one of the diagnostic criteria. Additionally, bone changes can impact vertebrae, leading to vertebral scalloping, and may be associated with conditions such as dural ectasia, scoliosis, and, less commonly, kyphosis. Vertebral dystrophic changes are often locally accompanied by paravertebral plexiform neurofibromas (PNs) [2, 3]. In NF1 patients, the presence of vertebral dystrophic changes and PNs makes surgical correction of spinal deformities challenging due to poor bone quality, complex anatomical structures, and severe spinal curvature [4].

There is limited literature on cervical kyphosis due to NF1, and no consensus has been reached on the best surgical approach for pediatric cases, even though these cases are rare. Treatment options include anterior-only (AO), posterior-only (PO), and combined anterior–posterior (CAP) approaches, but the choice of surgical strategy remains controversial and must be adapted to each patient’s unique situation, especially as long-term data and large patient studies are still lacking [5].

Here, we report a case of progressive cervical kyphosis associated with NF1 that well describes the complexity of this scenario, both from a clinical and surgical point of view. We also report the outcome of the therapy with an MEKi (trametinib) used for controlling the growth of adjacent PNs and used before the surgical approaches. We finally provide a systematic review of the available literature on the topic.

## Materials and methods

This review followed the guidelines outlined by the Preferred Reporting Items for Systematic Reviews and Meta-Analyses (PRISMA) statement [6]. The study sought to answer the following PICOS (population, intervention, comparison, outcomes) criteria:

- Population: children with NF1 presenting cervical kyphosis.
- Intervention: surgical procedures of any type.
- Comparison: none.
- Outcomes: severity and age of onset; surgical outcomes; complications; orthopedic and neurological comorbidities related to NF1.

A systematic literature search was conducted in four databases—PubMed, Cochrane, Scopus, and Medline—from 2000 to June 2024. Keywords combined using “AND” and “OR” included: “NF1\Kyphoscoliosis, Neurofibromatosis\Kyphoscoliosis, NF1\Cervical kyphoscoliosis, Neurofibromatosis\Cervical kyphoscoliosis, NF1\Cervical kyphosis, and Neurofibromatosis\Cervical Kyphosis.” No limitations on document type or publication date were applied.

Three independent reviewers (L.A.N., C.S., F.D.P.) screened all articles. In the first step, duplicate and irrelevant studies were removed. The second step involved screening titles and abstracts to confirm eligibility based on the inclusion criteria. In the final step, a detailed full-text review of selected articles was conducted to exclude those meeting exclusion criteria.

Inclusion criteria were as follows: 1) randomized controlled trials, cohort studies, case–control studies, or case series/reports; 2) studies written in English; 3) published in peer-reviewed journals; 4) titles or abstracts specifically referencing cervical kyphosis in pediatric NF1 cases; and 5) studies detailing surgical interventions for cervical kyphosis. Studies were excluded if they 1) lacked complete methodology or results; 2) were literature reviews without full case documentation; 3) involved NF1 or kyphosis onset > 20 years old (only cases < 20 years were included if mixed-age populations were studied); or 4) focused solely on preoperative evaluations without detailing surgical treatments.

## Case report

**Fig. 1:**
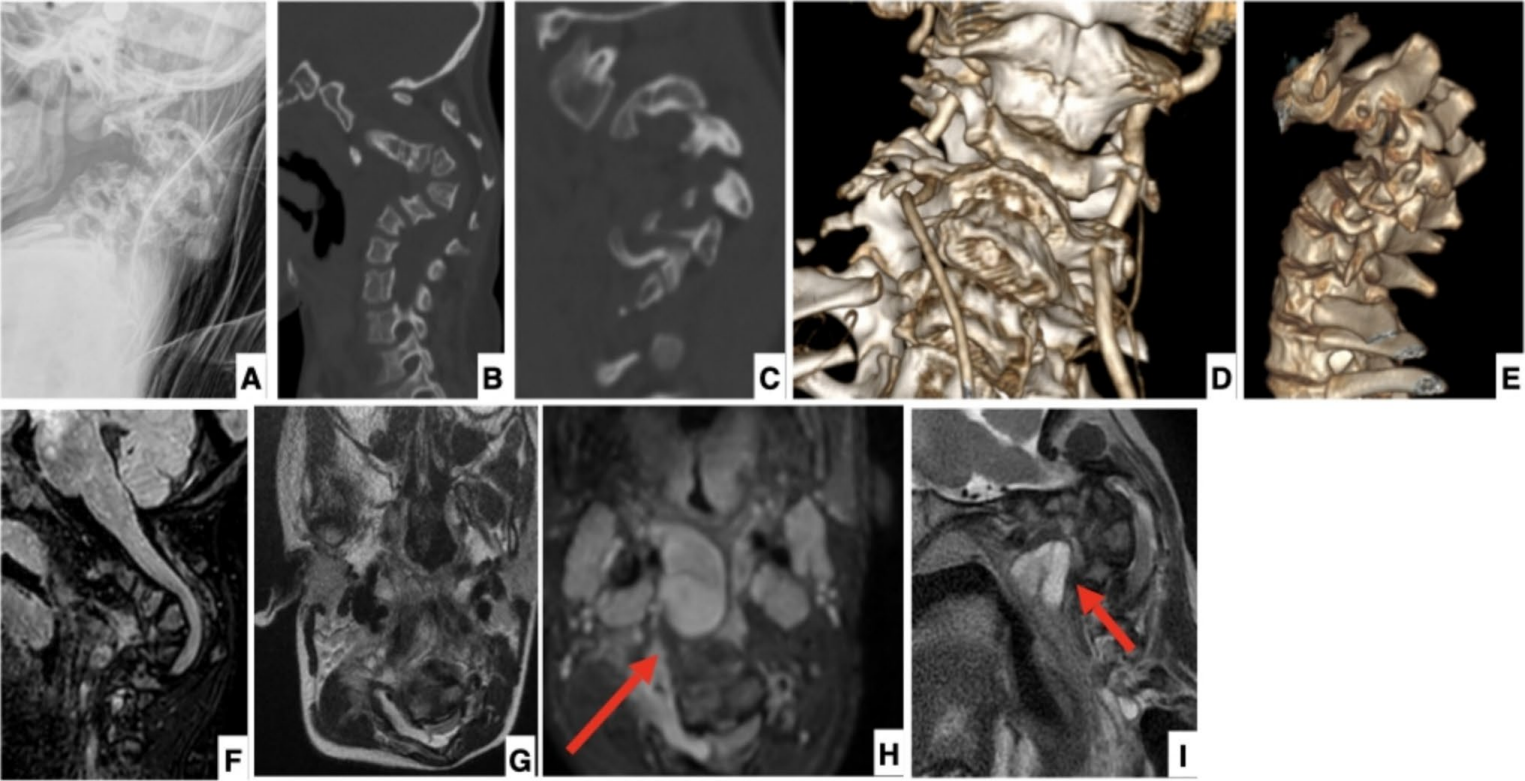
Imaging of the patient. **A** Pre-operative lateral view of the cervical spine. A severe kyphosis is demonstrated centered at C3. **B** CT scan reconstruction confirms the presence of dystrophic changes of the cervical vertebrae. **C** CT scan view of the right-sided facet joints shows dystrophic changes but no dislocation of the facets. **D** CT angiogram shows normal representation of the two vertebral arteries. **E** CT scan 3-D reconstruction of cervical spine deformity. **F** MRI scan shows tenting of the spinal cord around the kyphosis apex; the patient had no neurological deficits before surgery. **G** Axial image of the MRI confirms tenting and compression of the spinal cord at the apex of the kyphosis. **H** MRI axial image showing the extensive pre-vertebral neurofibromas (red arrow) encasing major blood vessels of the neck. The extensive neurofibromas present in the front of the spine made the anterior approach not feasible in this patient. **I** MRI sagittal view confirms the presence of extensive pre-vertebral neurofibromas (red arrow)

A 2-year-and-10-month-old girl was referred to our referral center because of a non-mobile and progressing swelling of the left retro-nuchal region, already present in the last 10 months, associated with café-au-lait macules. She is the only child of her parents. It was not possible to trace the father for any investigations. At the time of the visit, her height and weight were 96.8 cm (50° pc) and 14.8 kg (50° pc). Physical examination revealed the presence of typical NF1 café-au-lait macules, Noonan-like facial features, pectus excavatum, thoracic asymmetry, cervical spine kyphosis, relative macrocephaly, and disfigurement for head-neck asymmetry. This led to a strong clinical suspicion of NF1, and the patient underwent multi-specialistic clinical and instrumental examinations and tests. Blood tests, including bone metabolism, returned normal, and an echocardiogram showed only a mild to moderate mitral valve prolapse and minimal patent foramen ovale in the absence of signs of hemodynamic decompensation. Genetic tests confirmed the presence of a heterozygous deletion of approximately 14 Kb involving the first exon of the NF1 gene, which was not inherited by the mother (father not available for segregation study). The magnetic resonance imaging (MRI) of the brain and spine revealed an optic pathway glioma and right-convex cervico-thoracic scoliosis associated with severe cervical dysplasia and kyphosis. Plexiform neurofibromas (PNs) in the pre- and para-vertebral areas, arising from C2 to T3 on the right side, which encased major blood vessels of the neck, were also documented. The pathological tissue extended into the right C3–C4 and C4-C5 foraminal and in the posterior intracanal epidural site (from C3 to C5) (Fig. 1).

**Fig. 2:**
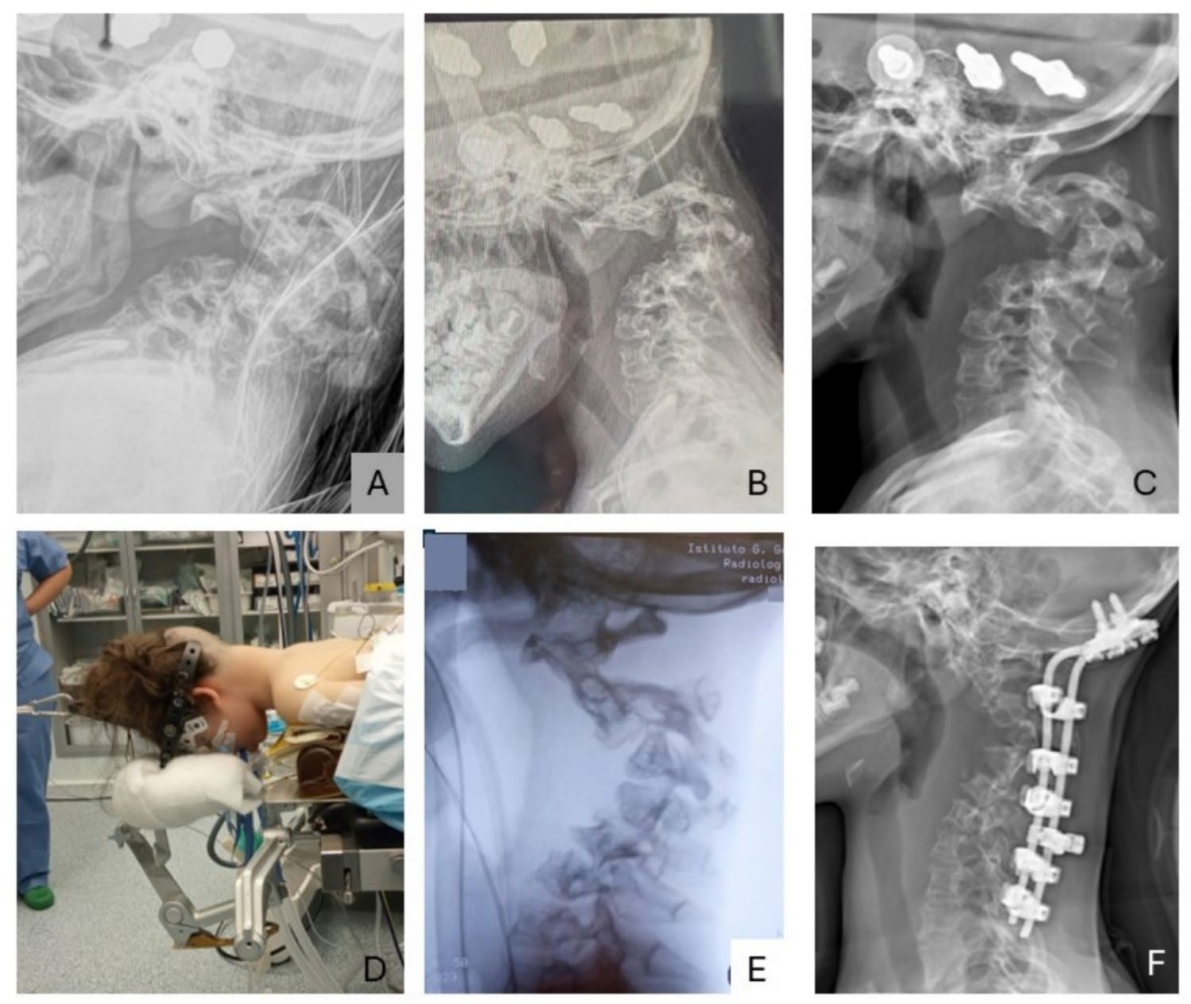
Progressive correction of the deformity. X-ray of the column before traction (**A**); soon after the traction procedure, an immediate reduction of the kyphosis was achieved (**B**); 6 weeks after traction (**C**). The patient is in traction on the surgical bed (**D**). Further traction under anaesthesia showed further improvement and correction of the kyphosis (**E**). Post-operative radiological X-ray (**F**)

An anteroposterior X-ray showed spinal dystrophy due to hypoplastic and dysmorphic vertebral bodies. Thus, an ophthalmological examination was performed too, resulting in substantially normal. After a multidisciplinary evaluation of the case, even if surgical stabilization of the cervical vertebra was recommended to prevent paraplegia and permanent neurological deficits, the vertebral column was considered not easily accessible for surgery due to the surrounding PNs. Complete surgical resection of the PN was considered not feasible, since the mass was too large and encased several anatomical structures. Since she was unable to swallow, no other treatments were available, so she started off-label therapy with Trametinib, an inhibitor of MEK1/2, at a dose of 0.5 mg per day when she was 3 years and 2 months old. The patient has been provided via the Novartis Managed Access Program. No side effects were observed except for eczematous dermatitis (grade 1) that occurred approximately 1 month after starting therapy and an increase in weight and body surface area, for which in July 2022 the dose was increased to 11 mg per day. After 2 years of follow-up, at 5 years old, cervical kyphosis continued to progress, and an X-ray documented a 170° Cobb angle on C3 (Fig. 2A). At that time, the patient had started to complain about swallowing difficulties, early satiety, and neck pain during her daily activities. On examination, the patient had no neurological deficits, although severe restriction of cervical spine range of motion (ROM) was noted. After a multidisciplinary reevaluation of the case, we decided to proceed with halo-gravity traction (HGT) prior to a surgical fixation of the cervical tract. Trametinib was stopped 2 days before the halo surgery. In the hospital, halo traction started with 0.5 kg and then weekly progressively increased to 5.5 kg (equal to 29% of the patient’s body weight) after 6 weeks (Fig. 2D). Traction was well tolerated by the patient with no complications. Progressive improvement in cervical kyphosis was observed with Cobb angle reduction from 170° to 90° at the end of the traction period (Fig. 2E). Interestingly the airway was scarcely visible before traction (Fig. 2A), and the HGT soon showed positive effect on it (Fig. 2C). The anatomy of the neck has been restored after surgery (Fig. 2G).

Furthermore, during the traction period, there was a significant reduction in feeding difficulties, resulting in a gain of 2 kg of weight. As the anterior approach to the cervical spine was deemed not feasible due to the extensive pre-vertebral neurofibromas, the decision was made to proceed with a single-stage posterior occipito-cervical fusion. During the posterior procedure, the patient was kept in traction on the surgical table; a standard posterior approach to the cervical spine was performed and the spine was fixed with screws and hooks. Further correction was achieved by segmental compression. Two standard titanium rods were used for fixation, and abundant bone graft was placed to promote fusion. The patient was kept in a halo brace after surgery for 3 months and then transitioned to a hard collar for three more months.

Pre-operative kyphosis was corrected to 69° Cobb, accounting for a 60% correction. The patient had no perioperative complications, and the wound healed with no signs of infection. The plexiform neurofibroma was visible on the left side of the neck, causing a disfigurement impact; the intraforaminal and intracanalar involvement was not worsened. Thus, in January 2024, Selumetinib was started providing through therapeutic nominal use by AstraZeneca, thanks to the fact that the patient was finally able to swallow, no adverse events have been observed till the latest cervical MRI of June 2024, which demonstrated stability of all known lesions. The last X-ray of the cervical spine was stable, too.

## Literature review

**Fig. 3:**
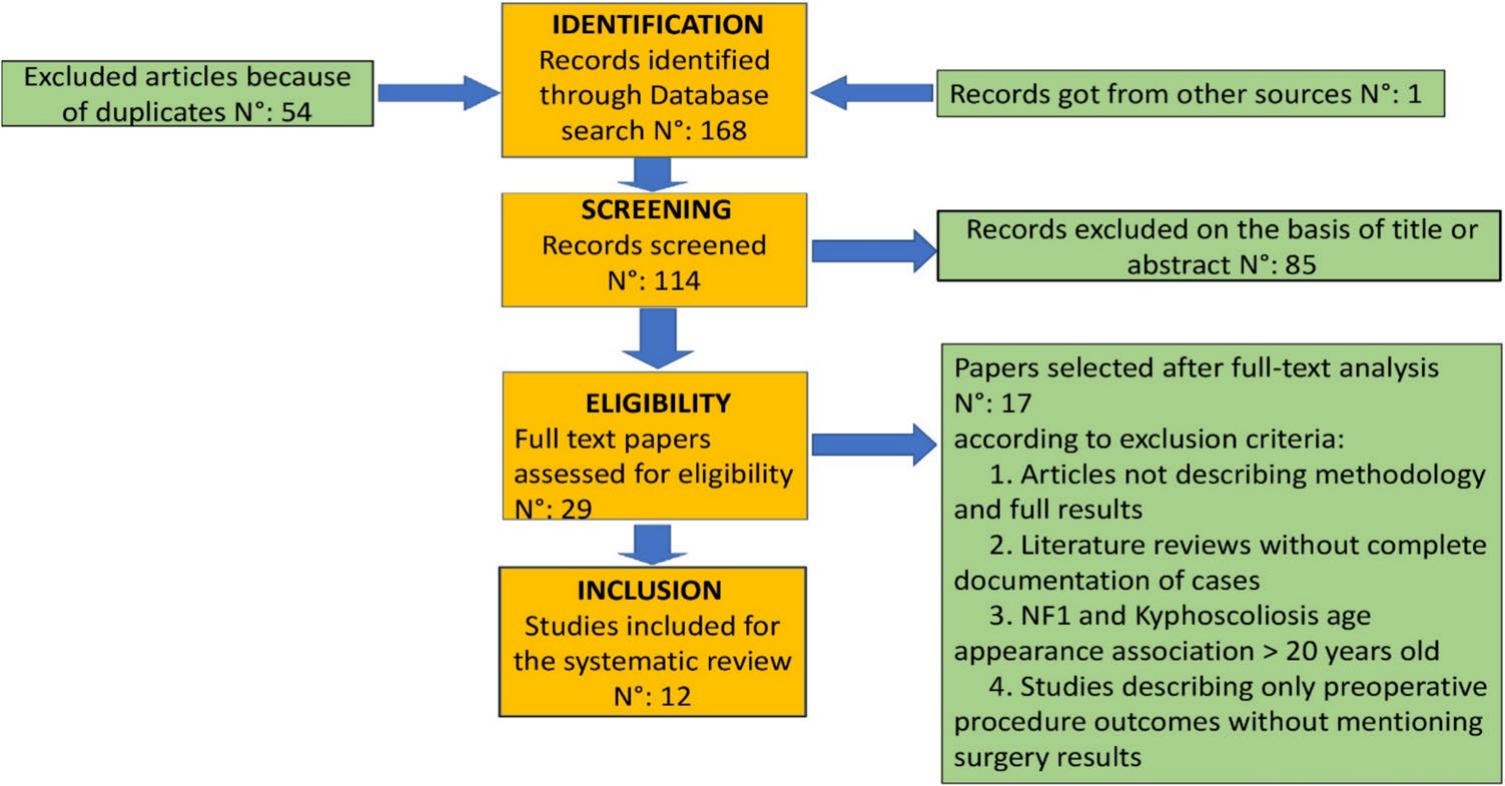
Literature review

Using the title word search, 239 references were found, and 83 references were selected because they were duplicates, resulting in 156 papers to examine. Furthermore, after analyzing their titles and abstracts, 106 references were excluded. Finally, after full text paper reading, 19 articles were selected to perform our final analysis, either for adulthood at onset (NF1 and kyphoscoliosis age appearance association > 20 years old) or for absence of correlation between NF1 and kyphoscoliosis or for describing only pre-operative procedure outcomes without mentioning any surgical intervention patients underwent with relative results or lack of full and complete documentation of cases. The search strategy and how the literature review was performed are shown in Fig. 3. We then used a structured form to record: main author and year of publication, sample size, follow-up, NF1 and kyphosis age appearance association, clinical presentation, surgery, pre-operative procedures, side effects, results measured in terms of local kyphotic angle and clinical improvements (Table 1). Among all studies analyzed for our systematic review, 10 were case reports [7–16], 3 case series [17–19], and 5 were retrospective studies [5, 20–23]. We took into consideration a total sample of 141 patients, 20 males and 12 females. Gender was not available in 109 patients [20–23]. The age at which the orthopedic diagnosis was made ranged from 11 months to 20 years.

**Table 1:** Clinical and surgical data of all reported children with NF1 and cervical kyphosis, together with our case

First author, year	Sample size (*n*)	Pre-operative procedures	Surgical intervention			Post-operative procedures	
		Halo gravity traction (*n*)	AO correction	PO correction	CAP correction and fusion	Halo vest	Philadelphia brace
Kawabata et al. 2013	3	3			3	3	
Vigneswaran et al. 2017	1	1			1	1	
Mladenov et al. 2020	6	6	1		5		
Wang et al. 2019	5	4			5		
Abe et al. 2003	1				1		
Gardner et al. 2009	1	Abandoned HGT			1		
Inoue et al. 2010	1			1			1
Kita et al. 2016	1			1			1
Lidar et al. 2012	1	1			1		
Li et al. 2024	6	6			6		
Wu et al. 2012	1	1		1			
Yifei et al. 2018	2	2	2				
Choksey and Modi 2015	1		1				
De Iure et al. 2014	1	1			1		
Helenius et al. 2016	22			9	13		
Stoker et al. 2012	1	1		1		1	
Ma et al. 2011	6		1		5		6
Lin et al. 2017	81	81	14	22	45		1
This case 2025	1	1		1		1	
Total *n* (%)	142	108 (76)	19 (13.4)	36 (25.3)	87 (61.3)	6 (4.2)	9 (6.3)

*AO*, anterior only; *PO*, posterior only; *CAP*, combined antero-posterior

There is no specific guideline for NF1-related PN management for whom surgery, watch-and-wait approach, and medical therapies can be considered, even in combination. Several MEK inhibitors (binimetinib, mirdametinib, trametinib) and the tyrosine kinase inhibitor (cabozantinib) have been used for NF1 complications, including PNs in childhood. Yet, the only drug with a specific indication for NF1 is selumetinib [24]. It is a selective inhibitor of MEK which has been approved by the US Food and Drug Administration in April 2020, and became reimbursable in Italy in 2020, for patients aged ≥ 3 years with neurofibromatosis type 1 (NF1) complicated by symptomatic, inoperable plexiform neurofibromas (PNs). It is administered orally, and the granulate suspension is not yet available. On the contrary, other drugs, for example, trametinib, have the advantage of formulation suitable for the youngest [25].

The treatment landscape has recently been deeply changed, yet the treatment choice in a child with a severe and complicated NF1 remains to be individualized, taking into consideration the age of the patient, the location of PN, its effect and growth rate, and other simultaneous complications.

All patients complained at least one clinical sign or symptom related to the cervical kyphosis which included intermittent neck pain (48 cases) [5, 16, 18–20, 23], shoulder girdle pain (1 case) [9], numbness of arms (6 cases) [16, 18, 19], gait disturbance (6 cases) [14, 15, 17, 18, 20], upper and\or lower extremity weakness (9 cases) [7, 8, 13–16], muscle paralysis whatever the type (60 cases) [12, 20], limitation of cervical range of motion (11 case) [5, 23], urinary dysfunction (1 case) [8], tactile and\or vibratory and\or pain sensibility dysfunction (2 cases) [8, 13], reduced rectal tone (1 case) [13], swallowing difficulty (1 case) [16], decreased bowel continence (1 case) [14]. Notably, the kyphosis was associated with scoliosis in 28 cases (19%) [8, 11, 14, 18, 20, 23]. Additionally, the patient described by Abe et all. [8] experienced an incomplete Brown–Séquard syndrome characterized by partial sensory loss below bilateral C5 dermatomes, muscle weakness in left upper and lower extremities, decreased tactile sensation below the C5 dermatome bilaterally, analgesia below the C6 level on the right side, diminished vibratory sensation on the left side and urinary dysfunction. One hundred three subjects (73%) were treated with pre-operative halo-gravity traction (HGT) for a variable period of time [5, 7, 13–15, 17, 18, 20–22], which improved cervical kyphosis angle in all cases except two who did not ameliorate at all [12, 15]. Seventy-six subjects (54%) underwent combined anterior and posterior cervical correction and fusion [5, 7–9, 12, 15, 16, 18, 20, 22, 23], 35 patients (25%) were treated with posterior-only cervical fusion [10, 11, 13, 14, 22, 23], and 24 patients (17%) with anterior-only spinal fusion [16, 17, 19, 21, 22]. Post-operative HGT was applied in 5 cases [7, 14, 18], and a Philadelphia brace was worn by the other 2 [10, 11]. Post-operative complications included wound infection in 9 cases (6%) [5, 9, 18, 22] and severe pneumonia in the other 6 (4%) [5, 18, 22] after CAP correction. One patient who underwent anterior fusion suffered from partial dislocation of the distal fibula graft after removing the halo vest [18].

In another patient, posterior wound swelling was noted 6 months after surgery; the wound was explored and found to be a subcutaneous mass of bone graft substitute. Once removed, the wound healed completely [5]. Finally, another case experienced perioperative complications such as leakage of cerebrospinal fluid, wound infection and severe pneumonia; for this reason, lateral ventricle trepanation and debridement were performed, and antibiotics were given with satisfactory results [5]. Subsequently, the patient was hospitalized for dysuria, a new episode of pneumonia, and asphyxia because of aspiration, for which he was transferred to the intensive care unit, where he died of multiple organ dysfunction syndrome.

Nine cases presented with junctional kyphosis [22, 23], in five cases a post-operative worsening of their neurological deficits was noted [23], and in another case a sudden weakness of the deltoid muscle [23]. In terms of clinical post-operative results, 109 patients achieved a global improvement of their neurological symptoms, as well as effective pain relief [9, 12, 15–18, 20, 21]. Additionally, 25 others achieved a full neurological recovery as their neurological physical examination demonstrated [8, 13, 14, 19, 23], and a stable neurological clinical picture was noted in 3 of them [7, 10, 11]. For the patients described by Lin et al., it was not possible to retrieve additional information [22].

## Discussion

Progressive cervical kyphosis is an uncommon complication of NF1 in pediatric patients. This condition is often severe and may result in spinal cord compression, potentially leading to paralysis. Early detection is crucial, with surgery remaining the primary treatment for progressive deformities. While various surgical techniques have been proposed to address severe and progressive cervical kyphosis in NF1, no universally accepted guidelines are currently available. According to existing literature, preoperative HGT aids in the initial correction of deformities, easing the subsequent surgical procedure. It also promotes muscle relaxation around the cervical spine and facet joints, which increases flexibility in rigid kyphosis cases, facilitating an anterior approach to the cervical spine [17–21].

Yifei et al. [17] suggest initiating traction at 4 kg, gradually increasing it to a maximum of one-eighth of the patient’s body weight. However, our case demonstrates that higher traction weights may be feasible, as we achieved traction at 30% of body weight. In our experience, regularly imaging the cervical spine during traction (e.g., lateral views to rule out occipito-cervical dislocation or subluxation) and slowly increasing weight (e.g., weekly) helps soft tissues adapt. Intraoperative traction can also be used to maintain neck extension and benefit from muscle relaxation during anesthesia, allowing additional correction. The AO approach was rarely reported; Yifei et al. [17] noted that after anterior decompression and reconstruction, neurological symptoms improved in all cases, with an average correction rate of about 83%. Although more extensive kyphosis correction is often associated with better neurological outcomes, the risks of nerve injury with aggressive correction remain significant, likely due to increased compressive forces at the kyphosis apex, spinal cord stretch, or nerve root tension during cervical lordosis restoration. Thus, an 80% correction rate is suggested as an adequate and safe target.

Choksey et al. and Ma et al. advise the anterior-only approach only for flexible or fixed deformities without facet joint ankylosis [16, 19]. They argue that the anterior approach allows better neural decompression and offers “superior surgical leverage” compared to posterior surgery. However, concerns exist about graft mobilization in anterior-only approaches due to the weaker fixation achieved, which is why anterior fusion is often combined with posterior fusion in NF1 cases [9]. Helenius et al. [18] compared outcomes between combined anterior–posterior (CAP) and posterior-only (PO) fusions, finding that patients treated with CAP had a higher correction rate for cervical kyphosis (83 vs. 58% in PO, *p* = 0.031). Additionally, CAP was associated with a lower risk of non-union in NF1 children, though complications such as junctional kyphosis and new temporary neurologic deficits affected more than half of the cases, with over a third requiring revision surgery [10]. Thus, fusion across more than five levels is recommended to minimize the risk of junctional kyphosis [18].

In the largest study to date, where patients were categorized based on the AO, PO and CAP approaches, Lin et al. [22] suggested that the CAP approach provided more stable and reliable correction of cervical kyphosis without further progression compared to AO or PO, with no significant differences found between the latter two approaches. However, as Li et al. [21] note, the final surgical approach selection depends on factors such as the degree of kyphosis, vertebral deformity extent, kyphosis curve flexibility, the specific location of nerve compression, and the presence of intraspinal or paravertebral plexiform neurofibromas (PNs). A detailed anatomical study of each patient is essential for an optimal surgical plan.

## Conclusions

In cases of progressive cervical kyphosis and NF1, surgical treatment is advised to prevent neurological signs and cervical pain. According to the available literature, HGT is well tolerated and safe in NF1 patients with cervical kyphosis, providing a significant amount of correction and making the following surgery easier. When feasible, anterior and posterior (circumferential) fusion is advised. Combined anterior and posterior fusion has been shown to provide the best outcomes in terms of fusion, kyphosis correction, and patient satisfaction. However, anterior or posterior surgery is not always feasible due to neurofibromas, soft tissue involvement, or the specific anatomy of the patient. Therefore, surgical plans should adapt to the specificity of the patient. Our case shows that a posterior-only approach can be a valuable alternative to a combined anterior–posterior approach. However, in case only posterior or anterior fusion is performed, the surgeon should keep a watchful eye on potential medium- and long-term complications (i.e., non-union and risk of instrumentation failure).

## Data Availability

Raw data for the manuscript will be made available upon request to the corresponding author of the manuscript.
